# Acetyl-11-Keto-Beta Boswellic Acid (AKBA) Protects Lens Epithelial Cells Against H_2_O_2_-Induced Oxidative Injury and Attenuates Cataract Progression by Activating Keap1/Nrf2/HO-1 Signaling

**DOI:** 10.3389/fphar.2022.927871

**Published:** 2022-07-11

**Authors:** Tianke Yang, Xiaolei Lin, Hongzhe Li, Xiyue Zhou, Fan Fan, Jianing Yang, Yi Luo, Xin Liu

**Affiliations:** ^1^ Department of Ophthalmology, Eye Institute, Eye & ENT Hospital, Fudan University, Shanghai, China; ^2^ NHC Key Laboratory of Myopia (Fudan University), Shanghai, China; ^3^ Key Laboratory of Myopia, Chinese Academy of Medical Sciences, Shanghai, China; ^4^ Shanghai Key Laboratory of Visual Impairment and Restoration, Shanghai, China

**Keywords:** acetyl-11-keto-β-boswellic acid, age-related cataract, Nrf2, oxidative injury, apoptosis

## Abstract

Age-related cataract (ARC) is one of the leading blinding eye diseases worldwide. Chronic oxidative stress and the apoptosis of human lens epithelial cells (HLECs) have been suggested to be the mechanism underlying cataract formation. Acetyl-11-keto-β-boswellic acid (AKBA) is a pentacyclic triterpene with antioxidative and antiapoptotic effects. In this study, we investigated the potential effects of AKBA on oxidative-induced HLECs injury and cataract formation. H_2_O_2_ was used to simulate HLECs oxidative injury *in vitro*, and Na_2_SeO_3_ was applied to establish an *in vivo* cataract model. In our current study, a cell counting kit-8 (CCK-8) assay was performed to evaluate the effects of H_2_O_2_ and AKBA on cell viability *in vitro*. Intracellular reactive oxygen species (ROS) levels were measured with the ROS assay to verify the antioxidant capacity of AKBA. Apoptotic cells were detected and measured by TUNEL staining and flow cytometry, and quantitative real-time (qRT)-PCR and Western blotting were applied to examine the transcription and expression of apoptosis-related proteins. Furthermore, immunofluorescence staining was performed to locate factor-erythroid 2-related factor 2 (Nrf2), and the protein levels of Nrf2, kelch-like ECH-associated protein 1 (Keap1) and heme oxygenase-1 (HO-1) were determined by Western blotting. Finally, we observed the degree of lens opacity and performed hematoxylin-eosin (H&E) staining to assess the protective effect of AKBA on cataract formation *in vivo*. AKBA increased HLECs viability under H_2_O_2_ stimulation, decreased intracellular ROS levels and alleviated the cell apoptosis rate *in vitro*. AKBA significantly decreased the expression of caspase-3 and Bax and increased the content of Bcl-2. The results of immunofluorescence and immunohistochemical staining proved that the expression and nuclear translocation of Nrf2 were activated with AKBA treatment *in vivo* and *in vitro*. Moreover, computational docking results showed that AKBA could bind specifically to the predicted Keap1/Nrf2 binding sites. After AKBA activation, Nrf2 dissociates from the Nrf2/Keap1 complex, translocates into the nucleus, and subsequently promotes HO-1 expression. In addition, AKBA attenuated lens opacity in selenite-induced cataracts. Overall, these findings indicated that AKBA alleviated oxidative injury and cataract formation by activating the Keap1/Nrf2/HO-1 cascade. Therefore, our current study highlights that AKBA may serve as a promising treatment for ARC progression.

## Introduction

Age-related cataract (ARC), characterized by lens opacification, is the leading cause of blindness and visual impairment worldwide (Khairallah et al., 2015; Lee and Afshari, 2017). Surgical treatment is currently the only effective option to manage cataracts. However, intraoperative and postoperative complications cannot be fully avoided, resulting in irreversible visual disability. On the other hand, the cataract surgery rate is relatively low in developing areas because of great socioeconomic burdens (Wang et al., 2017; Yan et al., 2019). Therefore, developing pharmacological treatment to prevent or delay cataract formation is crucially important.

The progressive accumulation of oxidative damage in the lens far exceeds the inherent antioxidant detoxification capacity, leading to crystallin aggregation and apoptosis of human lens epithelial cells (HLECs) (Liu et al., 2017). Therefore, strategies to restore and maintain redox balance for cataract prevention must be identified (Periyasamy and Shinohara, 2017).

The Keap1/Nrf2/HO-1 modulation system has long been recognized as a major defense mechanism for oxidative stresses (Liu et al., 2017). Nuclear factor erythroid 2-related factor-2 (Nrf2) is a vital nuclear transcription factor that binds to the antioxidant response element (ARE) in the DNA promoter and acts as a molecular switch that turns on/off the cellular redox balance systems (Mitsuishi et al., 2012). These activate a chain of events that eventually affect the cellular oxidative status and defense capability against oxidative stresses (Kansanen et al., 2013). Keap1 serves as an oxidative stress sensor and is also a major negative regulator of Nrf2. Under normal conditions, Nrf2 binds to Keap1, and basal levels of Nrf2 in the lens remain at a low level (von Otter et al., 2010; Cai et al., 2021). Overproduction of reactive oxygen species (ROS) leads to the accumulation of Keap1 and suppression of Nrf2 (Periyasamy and Shinohara, 2017). Activated Nrf2 translocates to the nucleus, where it binds to ARE and mediates the expression of antioxidant protein genes, including heme oxygenase-1 (HO-1) (de Freitas Silva et al., 2018). Previous studies have revealed that Nrf2/HO-1 serves as a powerful defense process to protect HLEC lines (SRA01/04) from H_2_O_2_-induced oxidative stress and apoptosis (Ma et al., 2018; Ran et al., 2021). Thus, novel antioxidative compounds modulating the Keap1/Nrf2/HO-1 pathway may be regarded as prospective pharmacological targets for cataract therapy.

Acetyl-11-keto-β-boswellic acid (AKBA) is a pentacyclic triterpenoid derived from *Boswellia serrata* (Ahmad et al., 2019). AKBA possesses versatile medicinal activities, including antioxidant, anti-inflammatory, anti-ulcer, antitumor, and immunomodulatory actions. It has been shown to have antioxidant effects in age-related and chronic conditions, such as type 2 diabetes, multiple tumors and Alzheimer’s disease (Efferth and Oesch, 2020; Wei et al., 2020; Siddiqui et al., 2021). Other studies also proposed that AKBA could prevent cell apoptosis by regulating the Nrf2/HO-1 signaling pathways and Bax and caspase-3 expression (Ding et al., 2015; Ahmed et al., 2020). However, whether AKBA treatment has protective effects on HLECs undergoing oxidative injury remains unknown. Therefore, the current study investigated the antiapoptotic and antioxidative effects of AKBA on H_2_O_2_-induced oxidative damage in HLECs and selenite-induced cataracts in rats. We also examined the role of the Keap1/Nrf2/HO-1 cascade in the pathogenesis of ARC.

## Materials and Methods

### Lens Epithelial Samples

The anterior lens capsules (*n* = 30) were removed from donors (free of ocular diseases, age range of 51–75 years) in the Shanghai Red Cross Eye Bank. The anterior lens capsule samples (*n* = 60) were obtained from age-related cataract patients (aged 50–80 years, free of other ocular diseases) who underwent cataract surgery in the Eye & ENT Hospital of Fudan University. The tissue collection protocol was approved by the Ethics Committee of EENT Hospital and adhered to the principles of the Declaration of Helsinki. Every three lens capsule specimens add up to one test specimen for quantitative real-time (qRT)-PCR.

### Cell Culture and Treatment

HLECs purchased from the Cell Bank of Chinese Academy of Science (Shanghai, China) were cultured in Dulbecco’s modified Eagle’s medium (DMEM, Gibco) containing 10% fetal bovine serum (FBS, Gibco) in an incubator with 5% CO_2_ at 37°C. To create the oxidative stress microenvironment, the cells were incubated with 0, 50, 100, 150, 200, and 250 μM H_2_O_2_ for 24 h. AKBA (MedChemExpress) was dissolved in dimethylsulfoxide (DMSO, Sigma–Aldrich) as a stock solution of 20 mM. In the group pretreated with AKBA, the cells were pretreated with different concentrations of AKBA (0, 2, 4, 8, 16, 32, 64, 128, and 200 μM) for 1 h before H_2_O_2_ treatment (Rajabian et al., 2020).

### Cell Proliferation and Toxicity Assay

The Cell Counting Kit-8 (CCK-8) assay (Dojindo) was performed to detect the effects of H_2_O_2_ and AKBA on cell viability at different concentrations. HLECs were incubated in 96-well plates for 12 h. After treatment with H_2_O_2_ or AKBA, 10 μL CCK-8 working solution was added to each well. After 2 h, the absorbance value was determined at a wavelength of 450 nm.

### Annexin V-FITC/Propidium Iodide (PI) Assay

The percentage of apoptotic cells was assayed using the annexin V/FITC apoptosis detection kit (MultiSciences) according to the manufacturer’s directions. After culturing with different concentrations of AKBA and/or H_2_O_2_, the cells were collected and washed with ice-cold phosphate-buffered saline (PBS). At room temperature, the cells resuspended in binding buffer (1x) were incubated with 5 μL FITC-labeled annexin V and 10 μL propidium iodide (PI) for 5 min in the dark. Apoptosis rates were assessed by flow cytometry (BD Biosciences).

### ROS Assay

The ROS levels in HLECs were detected with an ROS assay kit (Beyotime). Following H_2_O_2_ or AKBA interventions, cells were cultured in serum-free medium containing 2ʹ,7ʹ-dichlorofluorescein diacetate (1:1000) for 20 min at 37°C. The samples were washed with serum-free medium three times. The images of HLECs were obtained with a fluorescence microscope (Zeiss).

### TUNEL Assay

Apoptotic cells were measured using a TUNEL Apoptosis Assay Kit (Beyotime). After treatment (as indicated), HLECs cultured on glass coverslips were washed with PBS and then immersed in 4% paraformaldehyde for 30 min. After incubation with 0.3% Triton X-100 at 25°C for 5 min, the samples were incubated in TUNEL detection solution for 60 min in the dark. Cell nuclei were counterstained with DAPI (Beyotime), and the fluorescence images were observed with a fluorescence microscope.

### Immunofluorescence Staining

After washing three times with PBS, the cell samples seeded on coverslips in 24-well plates were fixed with paraformaldehyde for 15 min. Then, the cells were permeabilized with 0.3% Triton X-100 for 10 min. After incubation for 1 h in QuickBlockTM IF blocking solution (Beyotime), the samples were treated with primary antibodies against Nrf2 (1:100, Proteintech) overnight at 4°C. The next day, the slides were incubated with fluorescein isothiocyanate (FITC)-conjugated secondary antibody (1:200, Abcam) for 2 h at room temperature in the dark. The nucleus was stained with DAPI and observed using fluorescence microscope.

### Western Blot Analysis

After treatment with different agents, HLECs were washed with ice-cold PBS. Protein was extracted with radioimmunoprecipitation assay (RIPA; Beyotime) buffer. The nuclear protein was obtained using a Nuclear-Cytosol Extraction Kit (Byotime) according to the manufacturer’s protocols. After centrifugation at 14,000 rpm at 4°C, the supernatant was obtained. Protein concentrations were measured by a BCA kit (Beyotime). Twenty micrograms of protein were electrophoresed and transferred to a nitrocellulose membrane. After blocking with 5% nonfat milk, the membranes were incubated with the appropriate primary antibodies for 12 h at 4°C and then with secondary antibodies for 1 h at room temperature. Antibodies against *β*-actin (1:1000), Histone-H3 (1:2000), Nrf2 (1:1000), Keap1 (1:1000), HO-1 (1:1000), NOX-1 (1:1000), caspase-3 (1:1000), Bax (1:5000) and Bcl-2 (1:1000) were purchased from Proteintech (Shanghai, China). The protein signals were detected by the Tanon 5200 chemiluminescent imaging system (Shanghai, China), and quantitative analysis was performed by ImageJ (version 1.8.0).

### Animal Model

The experimental protocol involving animal research was authorized and conducted according to the Association for Research in Vision and Ophthalmology under the guidelines of the Animal Welfare Act. Healthy 8-day-old Sprague–Dawley suckling rats were obtained from the Laboratory Animal Center of the Eye & ENT Hospital of Fudan University. The suckling rats and their mothers were housed in clean, sterile, polypropylene cages.

Briefly, 24 rats were randomly divided into 4 groups, and Group I served as the normal control. Group Ⅱ: AKBA treatment group. Group III: Sodium selenite (SE, Na_2_SeO_3_) treatment group. Group Ⅳ: SE + AKBA treatment group. On postpartum days 11, 13, and 15, rat pups of Groups III and Ⅳ underwent injection of sodium selenite (20 μmol/kg body weight) to generate a cataract model (Zhang P. et al., 2022). In addition, Group Ⅱ and Group Ⅳ received AKBA intraperitoneally at doses of 20 mg/kg from day 11 to day 15 postpartum. The dose of AKBA was based on previous studies (Ding et al., 2015; Ahmed et al., 2020). Rat pups in the SE + AKBA group were administered AKBA 1 h prior to sodium selenite injection. On postpartum day 24, images of cataracts in pups were obtained under pentobarbital sodium anesthesia. All animals were euthanized after examination of cataract formation. Eye tissue samples were isolated and collected.

### Observation of Lens Opacity Formation

All rats were monitored daily for the development of cataracts. Cataract formation was scored according to the classification of Hiraoka and Clark (Hiraoka and Clark, 1995) and named as follows: Grade 0: normal transparent lens, Grade Ⅰ: initial sign of nuclear opacity, Grade Ⅱ: slight nuclear opacity, Grade Ⅲ: diffuse nuclear opacity with some cortical scattering, Grade Ⅳ: partial nuclear opacity, Grade Ⅴ: nuclear opacity, and Grade Ⅵ: mature cataract.

### Histological and Immunohistochemical Analysis

Rat eye samples were fixed in Davidson’s fixative and then incubated with a 10% buffered formalin solution, dewaxed in xylene and rehydrated in a series of graded alcohols. The eye samples were embedded in paraffin and then cut into 6 μm slides. Next, tissue sections were prepared for hematoxylin and eosin (H&E) staining. Images were obtained with a light microscope (Carl Zeiss, Germany). The histopathological fragments were graded according to the method of Mulhern et al. (Mulhern et al., 2006) as follows: Grade 0: normal, Grade 1: appearance of vacuoles, Grade 2: formation of homogenized area, Grade 3: disappearance of anterior epithelium, Grade 4: existence of lens fibers and homogenized area only.

The expression of Nrf2 in the lens samples was tested *via* immunohistochemistry. The tissue sections were dewaxed and hydrated prior to antigen retrieval. After blocking with serum for 30 min, the sections were incubated with primary antibodies against Nrf2 (1:100, Proteintech) for 12 h at 4°C and then with secondary antibodies for 30 min at room temperature. The chromogenic reaction was developed using a DAB Horseradish Peroxidase Color Development Kit (Beyotime), and immunohistochemistry-positive cells were dark brown.

### RNA Extraction and Quantitative Real-Time (qRT)-PCR

Total RNA was extracted by Beyozol reagent (Beyotime) according to the manufacturer’s protocol. The RNA integrity was determined and then reverse-converted into cDNA in accordance with the instructions using a reverse transcription kit (Takara). qRT–PCR was performed using a SYBR Green Premix Pro TaqHS qPCR kit (Accurate Biology) as previously described (Liu et al., 2018). The cycle threshold data of the target gene were normalized to *β*-actin levels and were calculated with the 2^−△△Ct^ method. The primer sequences used were as follows: *β*-actin: (F) 5′-GCT​CCT​CCT​GAG​CGC​AAG-3′ (R) 5′-CAT​CTG​CTG​GAA​GGT​GGA​CA-3′; Nrf2: (F) 5′-TTC​CCG​GTC​ACA​TCG​AGA​G-3′ (R) 5′-TCC​TGT​TGC​ATA​CCG​TCT​AAA​TC-3′; caspase-3: (F) 5′-AGC​GAA​TCA​ATG​GAC​TCT​GGA-3′ (R) 5′-GGT​TTG​CTG​CAT​CGA​CAT​CT-3′; Bax: (F) 5′-GGT​GGG​GTC​ATG​TGT​GTG​G-3′ (R) 5′-CGG​TTC​AGG​TAC​TCA​GTC​ATC​C-3′.

### Molecular Modeling

The structure data file (SDF) formats of AKBA were obtained from PubChem (https://pubchem.ncbi.nlm.nih.gov), and the recipient was retrieved from the Protein Data Bank database. For the somatic proteins Nrf2 and Keap1, PyMOL software (version 2.3.4) was used to perform protein structure preparation that involved the removal of water molecules and ligands (Adasme et al., 2021). Modification of the receptor protein, such as hydrogenation and charge balance, was carried out using AutoDockTools. Molecular docking research of receptor proteins and small molecule ligands was performed with AutoDock Vina. Finally, the binding affinity was evaluated.

### Statistics

The data were expressed as the means ± standard deviation (SD) of the mean from three independent experiments. Values of different groups were evaluated by one-way ANOVA with Tukey’s multiple comparisons test. GraphPad Prism version 7.0 software was used to conduct statistical analysis. A value of *p* < 0.05 was considered to indicate a statistically significant difference.

## Results

### Differential Keap1/Nrf2 Expression Between Normal and ARC Lens Capsules

**FIGURE 1:**
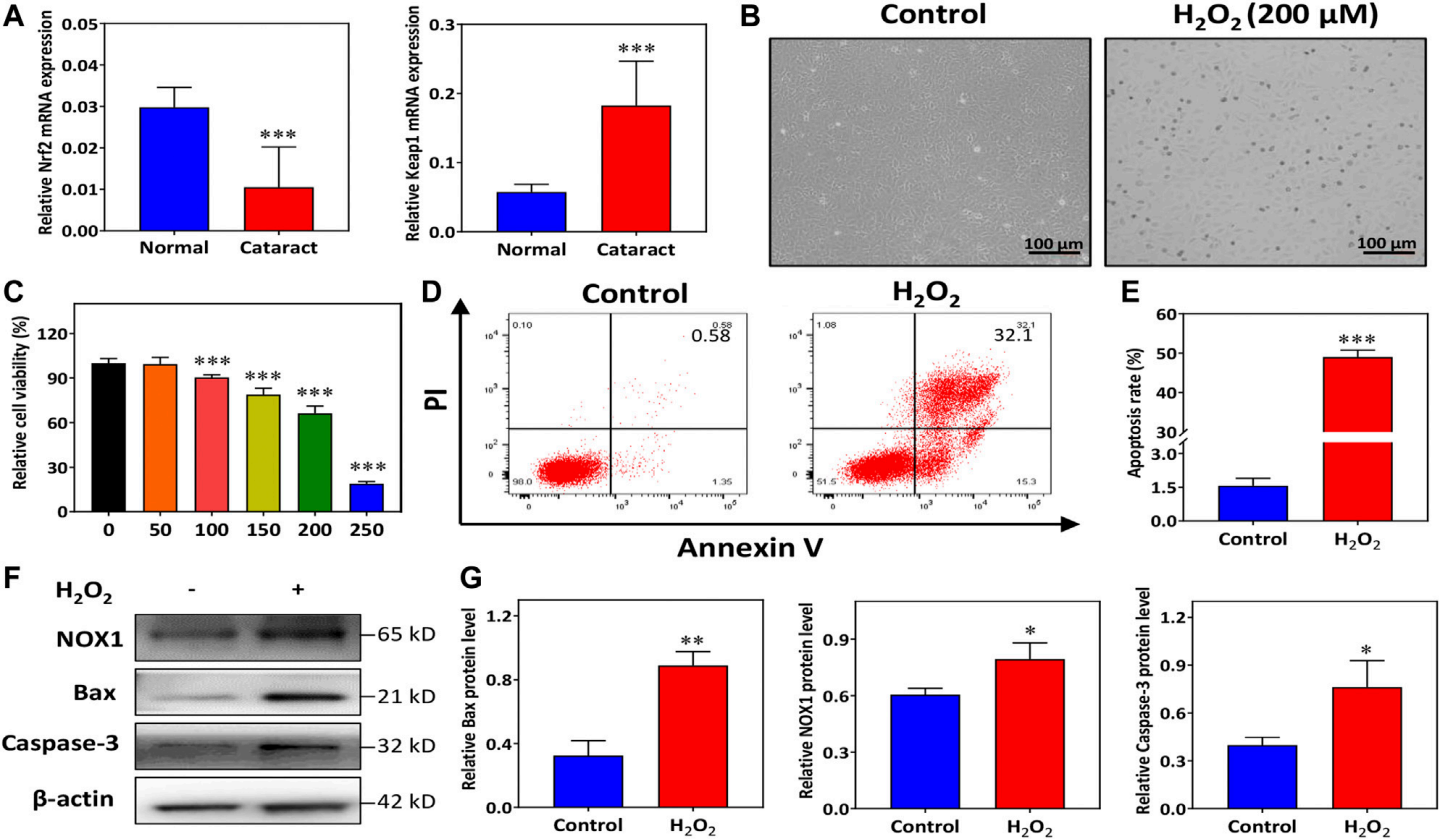
H_2_O_2_ promotes human lens epithelial cells (HLECs) apoptosis and oxidative injury. **(A)** The mRNA expression of Nrf2 and Keap1 in the capsule tissues of donors and cataract patients. **(B)** HLECs morphology was recorded with an inverted optical microscope. **(C)** The viability of HLECs treated with H_2_O_2_ for 24 h was assessed using a CCK-8 assay. **(D,E)** Flow cytometry analysis of total apoptosis induced by H_2_O_2_. **(F,G)** Western blot and quantitative analysis of NOX1, Bax and Caspase-3 protein expression levels in HLECs treated with H_2_O_2_ for 24 h. Data (*n* = 3) are represented as the mean ± SD. *p < 0.05; **p <0.01; ***p <0.001, vs. the control group.

It has been demonstrated that Nrf2 levels are decreased in human cataracts (Periyasamy and Shinohara, 2017). As expected, the results of Figure 1A show that the mRNA levels of Nrf2 were significantly downregulated in the capsule tissues of cataract patients compared with those of normal controls. Moreover, Keap1 was significantly upregulated in the cataract group compared with the normal control group (Figure 1A). Thus, the expression of Nrf2 was inhibited in human aging lenses.

### H_2_O_2_-Induced Cell Apoptosis and Oxidative Injury in HLECs

To construct oxidative stress conditions, HLECs were incubated with multiple concentrations of H_2_O_2_ (0, 50, 100, 150, 200, and 250 μM). Observation under an inverted microscope showed spindle shapes and even apoptosis-like changes, including rounding and shrinkage of cells before plasma membrane disruption, in most of the HLECs treated with H_2_O_2_ (Figure 1B). We observed that the viability of the cells was approximately close to the IC_50_ of H_2_O_2_ after treatment with 200 μM H_2_O_2_ for 24 h (Figure 1C). Therefore, we focused 200 μM H_2_O_2_ as the working concentration. HLECs apoptosis was determined with flow cytometry. Figures 1D,E demonstrated the results obtained in Annexin V-FITC/PI staining combined with flow cytometry, the percentage of total apoptotic cells was significantly increased in 200 μM H_2_O_2_ (Figure 1E).

To elucidate the potential molecular mechanisms involved in H_2_O_2_-induced oxidative injury, the levels of apoptotic and oxidative signaling molecules, including Bax, caspase-3, and NOX1, were detected. Western blotting results showed that compared to normal conditions, H_2_O_2_ induced overexpression of Bax and caspase-3 in HLECs (Figures 1F,G). The expression of NOX1 was markedly increased under H_2_O_2_ treatment (Figures 1F,G). These findings indicated that H_2_O_2_ treatment dramatically activated cell apoptosis and oxidative injury in HLECs.

### AKBA Treatment Attenuates H_2_O_2_-Induced Apoptosis and Oxidative Injury in HLECs

**FIGURE 2:**
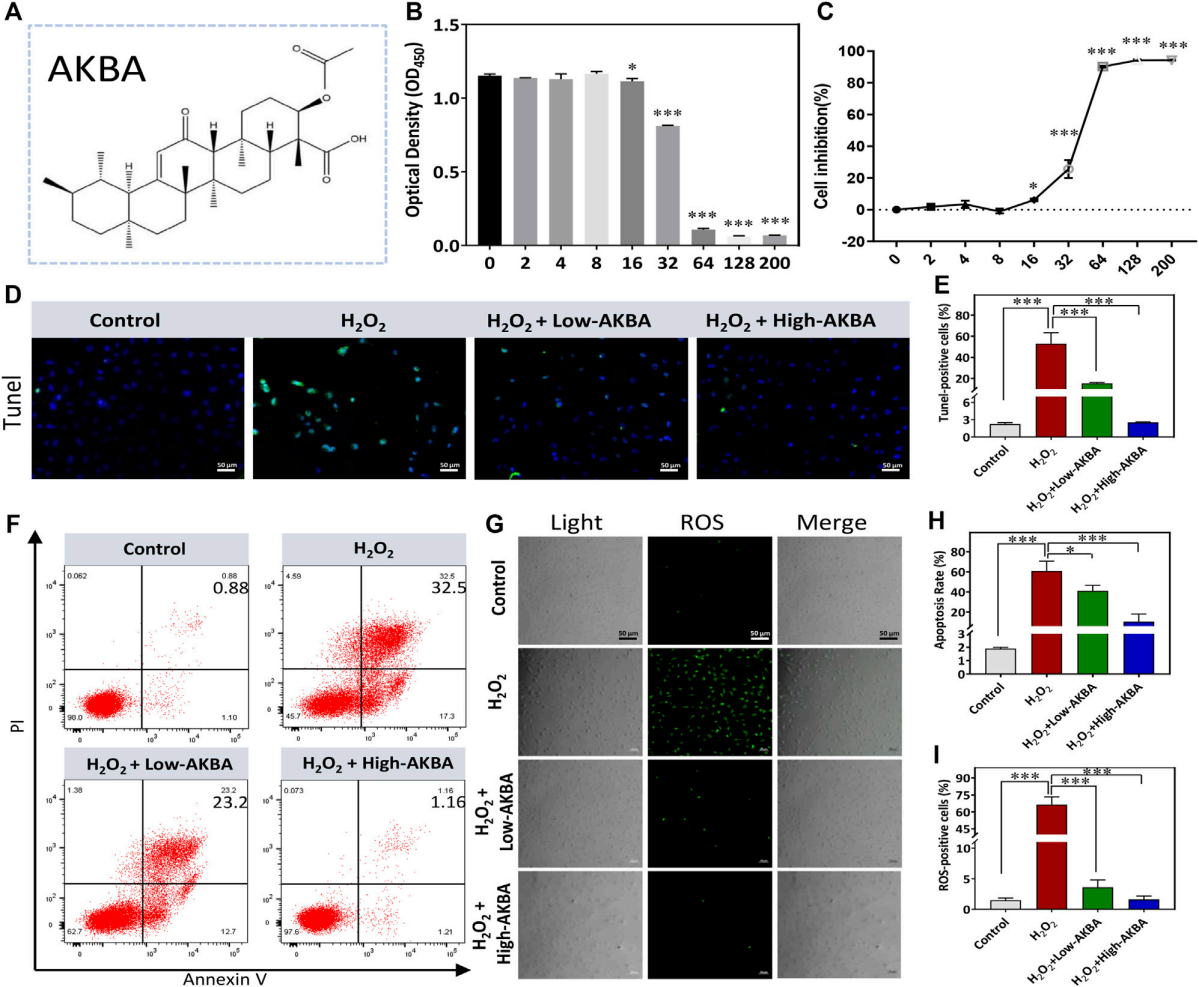
AKBA alleviates H_2_O_2_-induced oxidative injury and apoptosis in HLECs. **(A)** The chemical structure of AKBA. **(B)** Cell viability after 24 h of AKBA treatment was determined by CCK-8 assay. **(C)** Inhibition rate of HLECs. **(D)** TUNEL staining in HLECs. **(E)** Quantitative analysis of the TUNEL-positive cell ratio of each group. **(F)** Flow cytometry analysis of total apoptosis in the cells treated with H_2_O_2_, H_2_O_2_ + Low-AKBA (2 µM) and H_2_O_2_ + High-AKBA (8 µM) for 24 h **(G)** reactive oxygen species (ROS) staining in HLECs. **(H)** Quantitative analysis of total apoptosis rate in each group. **(I)** The percentage of ROS-positive cells per field in each group. Data (n = 3) are represented as the mean ± SD. *p < 0.05; ***p <0.001.

The chemical structure of AKBA is shown in Figure 2A. The CCK-8 assay was performed to determine the safe concentration range of AKBA on HLECs. Our results revealed that AKBA treatment at concentrations below 8 μM had no cytotoxic effects (Figures 2B,C). Thus, we chose 2 μM and 8 μM as low and high concentrations of AKBA to treat cells. As shown in the TUNEL assay results, a higher number of TUNEL-positive cells was observed in the H_2_O_2_-treated group than in the control group, and the number of apoptotic HLECs declined after AKBA treatment (Figures 2D,E).

To validate whether AKBA treatment could suppress H_2_O_2_-induced apoptosis in HLECs, we performed flow cytometry experiments to detect apoptotic cells. A relatively lower rate of apoptosis was observed after treatment with AKBA compared with H_2_O_2_ treatment alone. As shown in Figures 2F,H, the rate of apoptosis was greatly reduced as the dose of AKBA increased. Next, the oxidative injury caused by H_2_O_2_ treatment was investigated. The results suggested that the ROS level was significantly upregulated in HLECs treated with H_2_O_2_. Notably, the ROS levels in the AKBA-treated group were distinctly decreased compared with those in the H_2_O_2_-treated group (Figures 2G,I). Thus, AKBA treatment exerted a cytoprotective effect on HLECs under oxidative stress.

**FIGURE 3:**
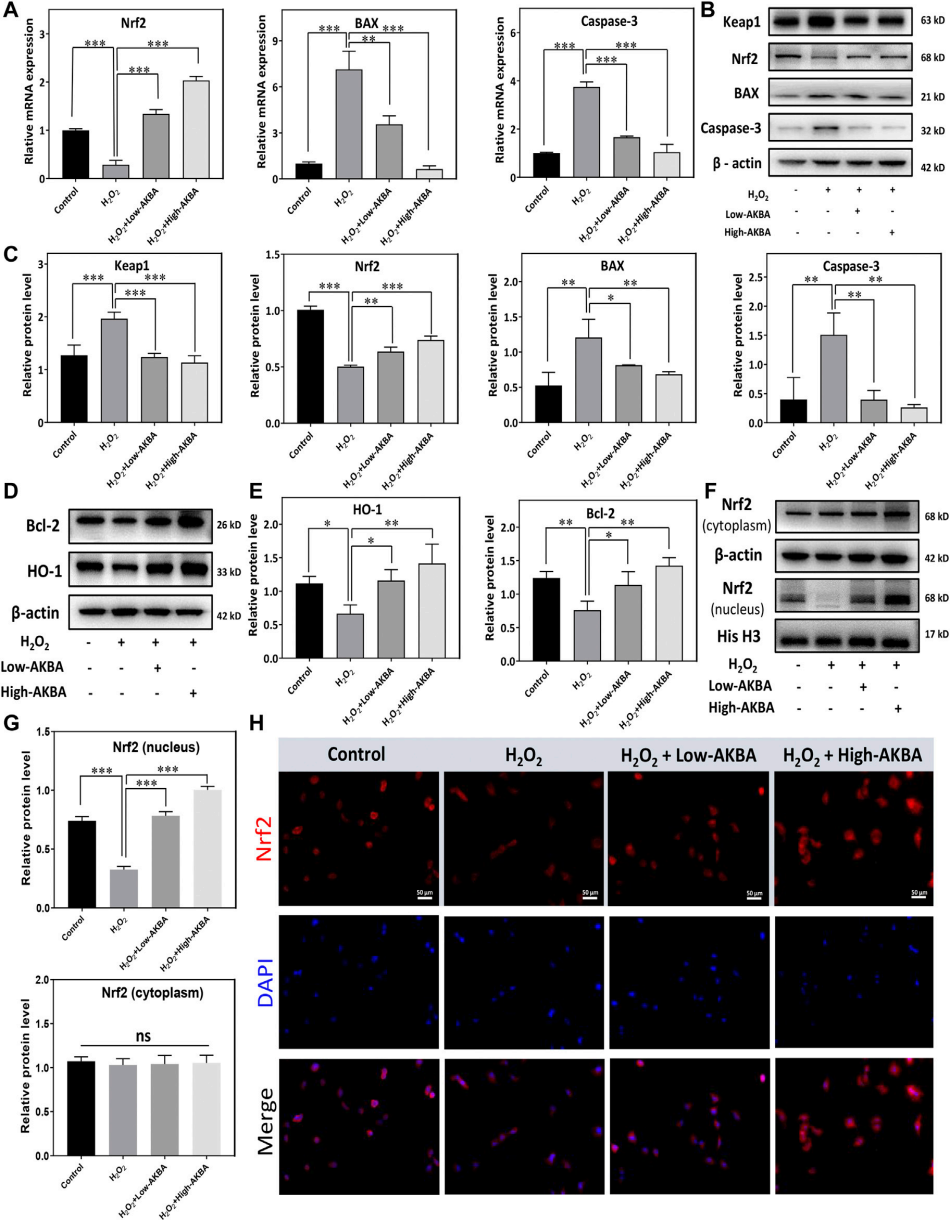
AKBA activates the Keap1/Nrf2/HO-1 signaling pathway in HLECs. **(A)** qPCR analysis of the mRNA levels of Nrf2 and apoptosis markers in HLECs treated with H_2_O_2_, H_2_O_2_ + Low-AKBA (2 µM), and H_2_O_2_ + High-AKBA (8 µM) for 24 h. **(B,C)** Western blot and quantitative analysis of Keap1, Nrf2 and apoptosis marker expression levels in HLECs treated with H_2_O_2_, H_2_O_2_ + Low-AKBA (2 µM) and H_2_O_2_ + High-AKBA (8 µM) for 24 h. **(D,E)** Western blot and quantitative analysis of HO-1 and Bcl-2 protein expression levels in HLECs treated with H_2_O_2_, H_2_O_2_ + Low-AKBA (2 µM) and H_2_O_2_ + High-AKBA (8 µM) for 24 h. **(F,G)** Western blot and quantitative analysis of nuclear and cytoplasmic Nrf2 protein expression levels in HLECs treated with H_2_O_2_, H_2_O_2_ + Low-AKBA (2 µM) and H_2_O_2_ + High-AKBA (8 µM) for 24 h. **(H)** Immunofluorescence image of Nrf2 in HLECs treated with H_2_O_2_, H_2_O_2_ + Low-AKBA (2 µM) and H_2_O_2_ + High-AKBA (8 µM) for 12 h. Data (*n* = 3) are represented as the mean ± SD. ns: no siginificance. *p < 0.05; **p <0.01; ***p <0.001.

To further demonstrate that H_2_O_2_-induced apoptotic signals were inhibited under AKBA treatment, hallmark apoptotic proteins were analyzed by western blotting. As shown in Figures 3A–E, qRT–PCR and western blot analyses showed that both low and high doses of the AKBA combination significantly reduced the expression of Bax and caspase-3 but upregulated Bcl-2 expression in HLECs after H_2_O_2_ stimulation. In combination, these findings indicated that AKBA ameliorated H_2_O_2_-induced oxidative injury and apoptosis in HLECs.

### AKBA Activates the Keap1/Nrf2/HO-1 Cascade in HLECs

The Keap1/Nrf2 signaling pathway is a major defense mechanism against oxidative stress. Activation of the Nrf2 pathway leads to the upregulation of cytoprotective enzymes, such as NAD(P)H:quinone oxidoreductase 1 (NQO1) and HO-1. It has been demonstrated that AKBA activates the Keap1/Nrf2/HO-1 signaling pathway (Wei et al., 2020). Therefore, we investigated whether AKBA regulates H_2_O_2_-induced apoptosis and oxidative injury in HLECs through the Keap1/Nrf2/HO-1 pathway. The results of the qPCR assay showed that Nrf2 mRNA levels were downregulated in the H_2_O_2_-treated group (Figure 3A). However, the Nrf2 mRNA level in the AKBA pretreatment group was higher than that in the H_2_O_2_-treated group (Figure 3A). In addition, western blotting showed that Nrf2 and HO-1 expression in HLECs was robustly decreased with H_2_O_2_ treatment, while Keap1 protein expression was upregulated (Figures 3B–E). Furthermore, our results illustrated that the Nrf2 and HO1 protein levels were significantly upregulated with AKBA treatment in HLECs after H_2_O_2_ stimulation, whereas the Keap1 protein was downregulated (Figures 3B–E).

We further investigated the correlation between Nrf2 translocation and AKBA activation. The western blotting results showed that Nrf2 translocated into the nucleus of HLECs treated with AKBA in a dose-dependent manner (Figures 3F,G). Additionally, fluorescence images revealed that Nrf2 was kept in the cytoplasm under normal circumstances. The Nrf2 protein was stained with a fluorescence-labeled antibody and is shown as red fluorescence. Nuclei were stained with DAPI and are shown as blue fluorescence (Figure 3H). As shown by western blotting and immunofluorescence staining, the nuclear localization of Nrf2 was increased in the AKBA-treated group (Figures 3F–H). Collectively, this evidence proved that the expression and nuclear translocation of Nrf2 were damaged when HLECs were exposed to H_2_O_2_. AKBA treatment alleviated the inhibition of the Keap1/Nrf2/HO-1 cascade caused by H_2_O_2_.

### Molecular Docking Between AKBA and Keap1/Nrf2

**FIGURE 4:**
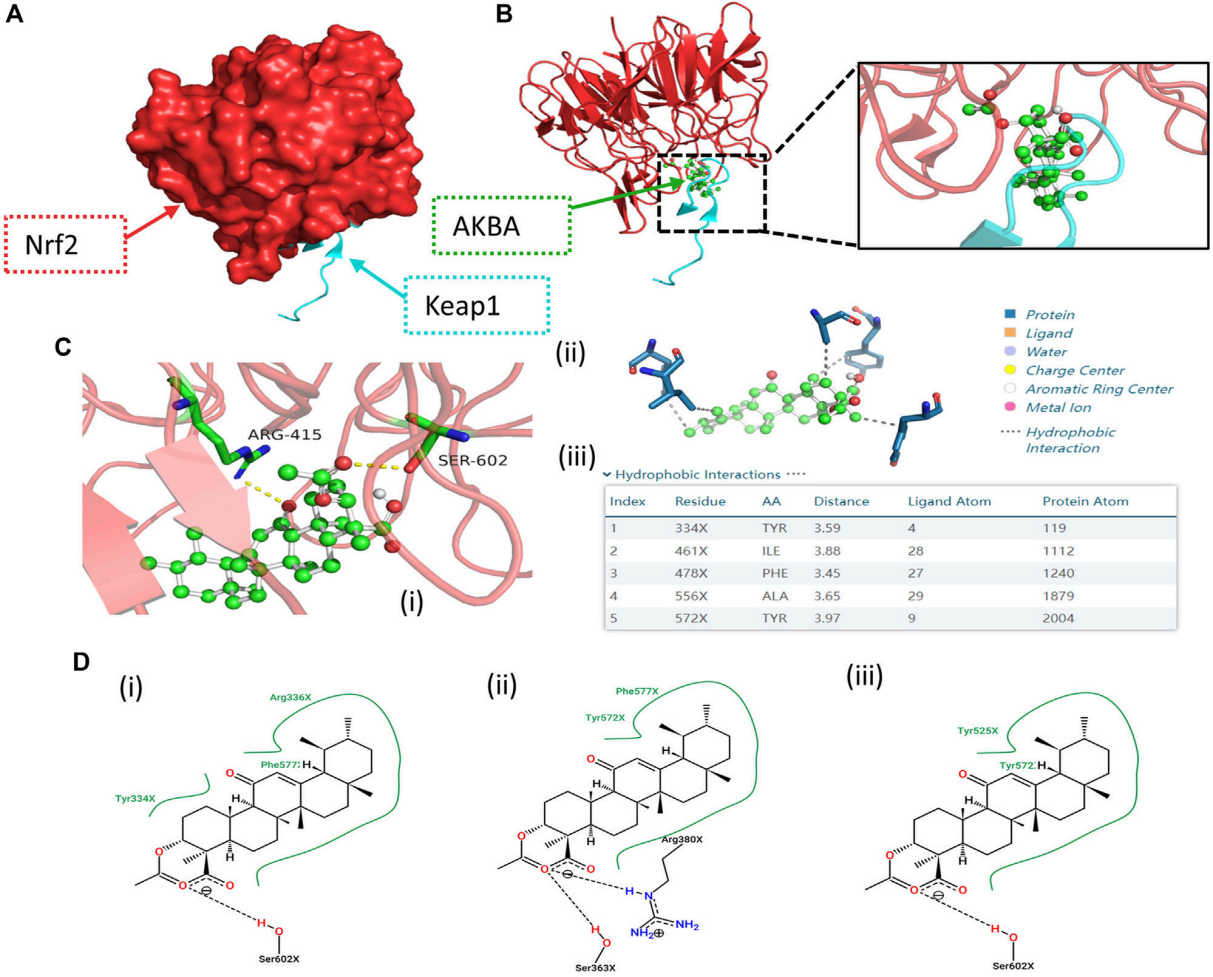
Computational docking results of the AKBA-Keap1/Nrf2 interaction. **(A)** Structural 3D image of the Keap1/Nrf2 complex. **(B,C)** Image of the secondary structure showing the interaction of Keap1/Nrf2 and AKBA with the lowest binding affinity. (i) Green region: AKBA; blue region: amino acids involved in bonding; yellow line: hydrogen bonds with polar atoms. (ii) Gray line: hydrophobic interaction. (iii) Top five molecular conformations with lower binding affinity. **(D)** Molecular conformations formed by Keap1/Nrf2 and AKBA with the (i) second, (ii) third and (iii) fourth lowest binding affinities, respectively.

Next, we tested whether there is any affinity between AKBA and Keap1/Nrf2 protein via a computational approach based on a combination of molecular docking. We constructed a crystal model of the Keap1/Nrf2 complex (Figure 4A). A variety of AKBA-Keap1/Nrf2 binding conformations were obtained by semiflexible docking, showing that AKBA has good binding to the Keap1/Nrf2 complex. Then, the binding affinity of these binding conformations was scored and ranked using AutoDock Vina. The top five binding conformations with lower binding energies were selected to visualize the prediction of the binding mode of Keap1/Nrf2 and AKBA (Schoning-Stierand et al., 2020; Adasme et al., 2021). Hydrophobic interactions and hydrogen bonds between AKBA and Keap1/Nrf2 were observed in the computational docking procedure (Figures 4B,C). In addition, the molecular conformations formed by Keap1/Nrf2 and AKBA with the second, third and fourth lowest binding affinities are shown in Figure 4D. The results demonstrated that AKBA interacts with and docks at the Nrf2-binding site on Keap1 and competes with Nrf2, resulting in Nrf2 nuclear translocation. Therefore, these data indicated that AKBA probably inhibited H_2_O_2_-induced oxidative injury and apoptosis by interacting with Keap1/Nrf2 and promoting Nrf2 nuclear translocation.

### AKBA Alleviated Lens Opacity in Selenite-Induced Cataracts in Rats

**FIGURE 5:**
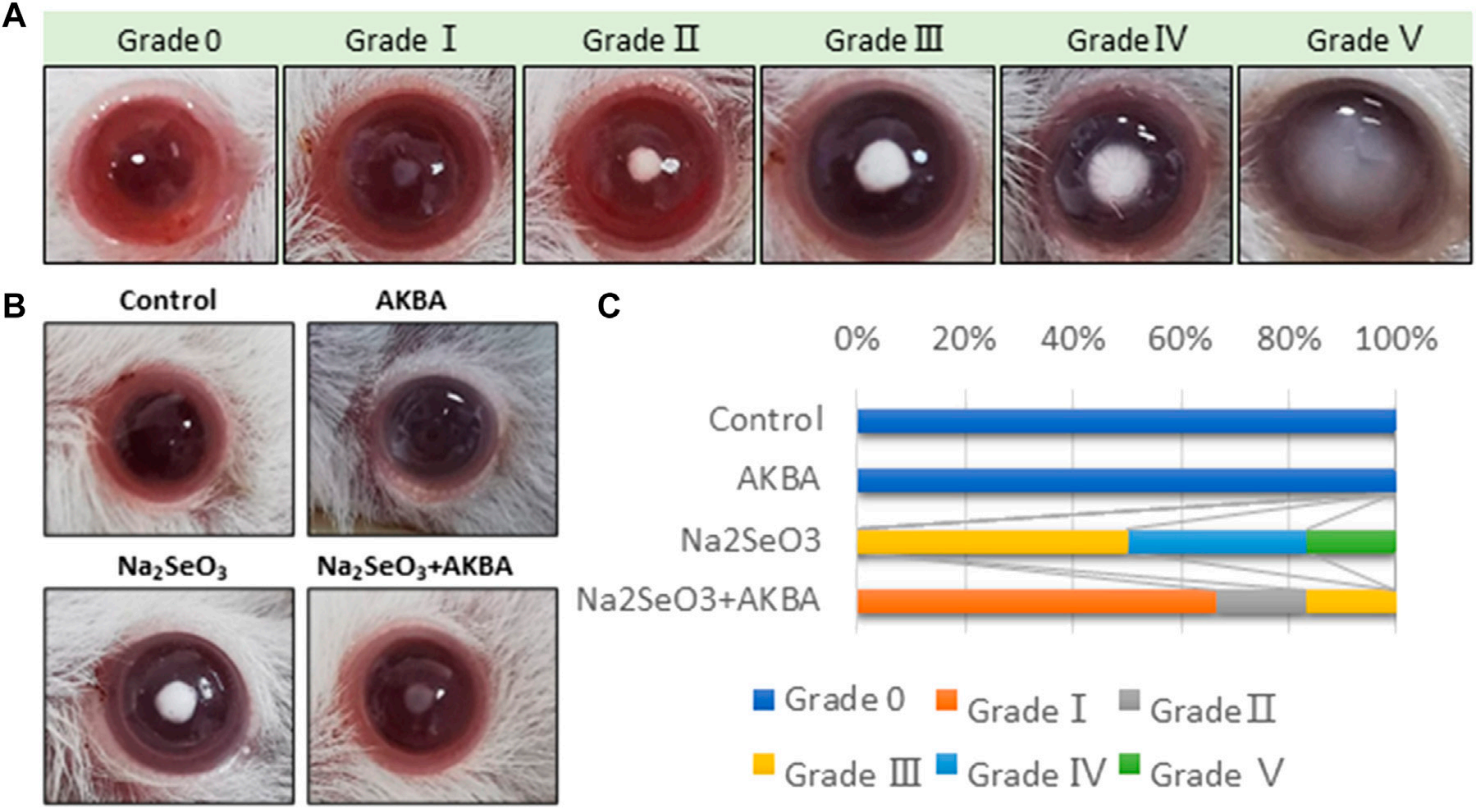
AKBA alleviates lens opacity in selenite-induced cataracts in rats. **(A)** Lens opacification was graded on a scale of Grade 0 to Grade V. **(B)** Lenses from the normal control group (Grade 0), AKBA treatment group (Grade 0), Na_2_SeO_3_ treatment group (Grade Ⅲ) and AKBA + Na_2_SeO_3_ treatment group (Grade Ⅰ). **(C)** The distribution of lens opacity grades in each group (*n* = 6).

To further validate the preventive effect of AKBA on cataract formation *in vivo*, we conducted intraperitoneal injection of AKBA in a rat selenite-induced cataract model. The lenses of rat pups in the normal control group and the AKBA group showed complete transparency. All Na_2_SeO_3_-treated rats showed cataracts with different degrees of opacity. However, the Na_2_SeO_3_-treated AKBA-pretreated pups exhibited mild cataracts. Lens opacification was graded on a scale of Grade 0 to Grade V. Photographs of representative cataracts are shown in (Figure 5A). The morphological score of lens opacification in the AKBA + Na_2_SeO_3_ group was remarkably lower than that in the Na_2_SeO_3_ group (Figure 5B). The distribution of lens opacity grades in each group is shown in Figure 5C.

**FIGURE 6:**
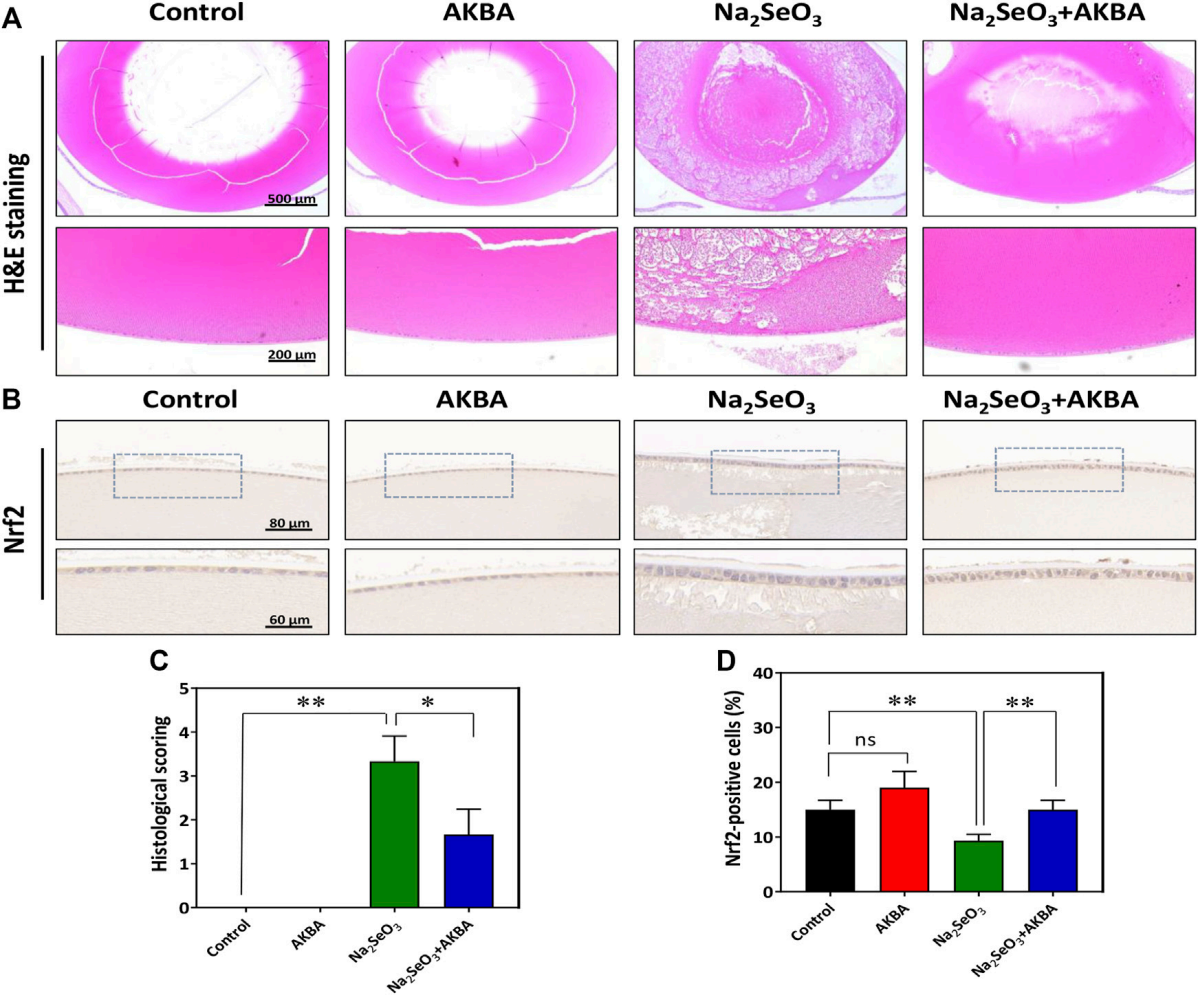
AKBA activates Nrf2 expression in lens epithelial cells and ameliorates histopathological alterations *in vivo*. **(A)** H&E staining images of the lenses from the normal control group, AKBA treatment group, Na_2_SeO_3_ treatment group and AKBA + Na_2_SeO_3_ treatment group. **(B)** Immunohistochemistry analysis of Nrf2 expression. **(C)** Quantitative histopathological score analysis of the lenses. **(D)** Quantification of the number of positive cells. *n* = 3 per group. Data are represented as the mean ± SD. ns: no siginificance. *p < 0.05; **p <0.01.

In this study, H&E staining was used for histological evaluation of rat eye samples. In the normal control group and the AKBA group, the lenticular epithelium and lens fibers remained intact, and no significant pathology was observed. Notably, histological analysis revealed obvious changes in the structure of the lens in the Na_2_SeO_3_ group. The lens showed diffuse cataract changes, including lens epithelial proliferation and degeneration or liquefaction of lens fibers. Administration of AKBA significantly ameliorated all of these lens alterations. Quantitative histopathological score analysis of the lens revealed a significant increase in the lesion score in Na_2_SeO_3_-treated pups (Figures 6A,C). However, the histopathologic score of the AKBA + Na_2_SeO_3_ group was 1.6 ± 0.6, which was notably lower than that of the Na_2_SeO_3_ group (3.3 ± 0.6). The number of immunohistochemistry-positive lens epithelial cells in the Na_2_SeO_3_ group was remarkably lower than that in the other groups. In addition, immunohistochemical staining revealed that AKBA significantly upregulated the expression of Nrf2 in lens capsule tissues (Figures 6B,D). Collectively, our data illustrated that AKBA inhibited the development of cataracts and increased Nrf2 expression in lens epithelial cells in rats.

## Discussion

The imbalance between oxidative stress and antioxidant protection contributes greatly to the onset and progression of ARC. Oxidation influences the protein solubility of the lens directly and progresses with age (Cekić et al., 2010). Thus, it is of vital importance to look for novel pharmaceuticals with antiapoptotic and antioxidant efficacy that could regulate cascade pathways and prevent ARC progression. Plant-derived Boswellia serrata, particularly the pentacyclic triterpenoid AKBA, has garnered great attention because of its assumed safety, apoptotic effect, and antioxidant therapeutic capacity (Ahmad et al., 2019). It has been described to activate the Nrf2/HO-1 pathways that are involved in the modulation of numerous cytoprotective and antioxidative genes (Minj et al., 2021). In addition, it has been suggested that AKBA exerts the antiangiogenic effects in a model of pathologic retinal neovascularization (Lulli et al., 2015). Thus, the current study was performed to determine the protective effect of the transcriptional inducer Nrf2 in ARC. Considering the beneficial properties of triterpenoid and its protective role through activation of the Keap1/Nrf2/HO-1 cascade, we also used *in vivo* and *in vitro* models to identify the role of AKBA in lens epithelial cells. We hypothesized that AKBA would provide antiapoptotic and antioxidant effects induced by Na_2_SeO_3_ in rats and H_2_O_2_ in HLECs *via* the Nrf2/HO-1 cascade. AKBA may serve as a new therapeutic agent to prevent or slow ARC progression.

The Keap1/Nrf2 signaling pathway is a powerful cellular defense system against oxidative damage. As an important nuclear transcription factor, Nrf2 modulates the transcription of various antioxidant genes by binding to antioxidant response elements (AREs) in DNA promoters, thereby maintaining redox homeostasis (Cullinan and Diehl, 2004). The expression of Nrf2 and downstream target genes exhibited an age-dependent reduction in rats (Shih and Yen, 2007). Total and nuclear Nrf2 expression were dramatically reduced in ARC lenses and diabetic cataractous lenses, illustrating that Nrf2 plays an antioxidant role during aging (Periyasamy and Shinohara, 2017). The demethylation of the Keap1 promoter significantly increased Keap1 expression and further activated Nrf2 proteasomal degradation (Periyasamy and Shinohara, 2017). Separation and deterioration of Nrf2 through Keap1 binding leads to inactivation of Nrf2-dominated protection and to progression of ARC (Gao et al., 2015). We also confirmed that Nrf2 was downregulated and Keap1 was upregulated in lens capsules with ARC compared to the normal control. Upon activation of various stress stimuli, such as oxidation, Keap1 undergoes conformational changes, which stimulate Keap1-Nrf2 complex separation and allow Nrf2 to translocate into the nucleus (Siddiqui et al., 2021). These data indicate that activation of Nrf2 might alleviate stress and slow aging, prompting research into developing anticataract formation compounds. Sodium selenite (Palsamy et al., 2014a), lack or excessive O_2_ and hypoglycemia under hypoxia (Elanchezhian et al., 2012) are known as Nrf2 suppressors, which accelerate cataract development by inhibiting the Nrf2 pathway in HLECs. Hyperoside (Park et al., 2016), acetyl-l-carnitine (ALCAR) (Yang et al., 2015) and sulforaphane (SFN) (Liu et al., 2013) are known as Nrf2 activators that activate Nrf2 and protect lenses against oxidative damage (Liu et al., 2017). These findings further demonstrate that Nrf2 is a prospective pharmaceutical agent for preventing and delaying ARC progression.

The present study probes the mechanisms by which AKBA reduces apoptosis and oxidative stress and promotes Nrf2 translocation into the nucleus. To test our hypothesis, we first evaluated the effect of AKBA on HLECs following H_2_O_2_ treatment and found that AKBA treatment significantly attenuated cell apoptosis by modulating the Bax/caspase-3 pathway. Furthermore, we discovered that AKBA inhibited ROS formation.

AKBA protects HLECs against H_2_O_2_-induced apoptosis. Exogenous H_2_O_2_ treatment is a classic and feasible cell model that can effectively simulate the oxidative damage process in HLECs, which could eventually lead to cataract formation (Li et al., 2019). In this study, H_2_O_2_ induced HLECs apoptosis. Under acute oxidative stress (200 μM H_2_O_2_), HLECs experienced a morphological change with rounding and cell shrinkage. Additionally, AKBA reduced the apoptosis of HLECs treated with H_2_O_2_. The caspase family and the Bcl-2 family both regulate HLECs apoptosis (Ran et al., 2021). The mitochondrial membrane protein Bcl-2 regulates the translocation of cytochrome C and inhibits the activation of caspase, which determines the direction of apoptosis pathways (Czabotar et al., 2014). Bax and Bcl2 serve as apoptotic and antiapoptotic markers, and the ratio of their proteins is a vital indicator of cell apoptosis (Fang et al., 2012). In HLECs, our data demonstrated that Bax and caspase-3 expression was dramatically upregulated, while Bcl-2 expression was notably downregulated following H_2_O_2_ treatment. These apoptotic proteins were dramatically downregulated after AKBA treatment in a dose-dependent manner. These results demonstrate that AKBA can protect HLECs from apoptosis caused by oxidative stress.

AKBA alleviates H_2_O_2_-induced oxidative damage by reducing the activity of ROS. The impairment of homeostasis between oxygen free radicals and antioxidant systems is considered a key molecular mechanism for the onset and progression of ARC (Cekić et al., 2010). H_2_O_2_-induced oxidative injury increased ROS production and blocked the antioxidative system, which accelerated the aggregation of free radicals (Bai et al., 2013). It has been reported that AKBA seems to reduce lipid peroxidation and restore antioxidant enzymes like SOD through Nrf2/HO-1 pathway (Rajabian et al., 2020). In our study, AKBA-pretreated HLECs observably suppressed intracellular ROS levels after H_2_O_2_ incubation, resulting in higher cell viability in HLECs. The level of ROS in the H_2_O_2_-treated group was significantly higher than that in the group pretreated with a high dose of AKBA. A previous study demonstrated that AKBA has the protective capability that suppresses NOX1 activity, a major mediator of oxidative-derived ROS generation (Yang et al., 2017). The NOX family is a series of key molecules involved in cellular oxidative responses (Brandes et al., 2014). Activation of NOX1 causes excessive production of superoxide, which leads to cell apoptosis (Brewer et al., 2015). Combining the ROS scavenger role of AKBA, our study indicates that AKBA has a protective effect on HLECs by reducing oxidative injury and subsequent apoptosis.

To confirm the underlying mechanisms of AKBA in HLECs, we examined whether AKBA regulates H_2_O_2_-induced apoptosis and oxidative injury through the Keap1/Nrf2/HO-1 signaling pathway. Accumulating evidence has indicated that Nrf2/HO-1 regulation is strongly correlated with the development and progression of several biological disorders (Mao et al., 2022; Yang et al., 2022; Zhang R. et al., 2022). Nrf2/HO-1 activation protects HLECs from H_2_O_2_-induced apoptosis and oxidative and ER stress (Ma et al., 2016; Ma et al., 2018). H_2_O_2_-induced cell oxidative injury is known to activate Nrf2 phosphorylation and nuclear translocation, leading to the activation of a variety of antioxidant and detoxification enzymes, such as HO-1, NQO1 and GST (Jiang et al., 2017; Yang et al., 2017; Zhou et al., 2019). These enzymes are crucial for reducing and eliminating oxidative injury and apoptosis, resulting in endogenous redox balance (Jian et al., 2011; Zhou et al., 2019; Yang et al., 2022). We show that Nrf2 and HO-1 levels decrease in HLECs due to H_2_O_2_ treatment, which is consistent with previous research (Palsamy et al., 2014b). Activation of the Keap1/Nrf2/HO-1 signaling pathway and its subsequent restoration of antioxidant and antiapoptotic proteins was associated with AKBA treatment in a dose-dependent manner. As shown in the figures and molecular docking, treatment with AKBA activated the Keap1/Nrf2 cell signaling pathway targeting HO-1. AKBA stimulated the nuclear aggregation of Nrf2 and reduced Bax levels, therefore promoting HO-1 expression. Overall, AKBA protects HLECs from H_2_O_2_-induced oxidative injury mainly by regulating Nrf2 translocation-driven proteins and therefore leads to suppression of oxidation and apoptosis. These findings strongly suggest that AKBA elicits Keap1/Nrf2/HO-1-mediated antioxidant and antiapoptotic responses.

Moreover, we also examined the effects of AKBA on the progression of ARC in a Na_2_SeO_3_-induced cataract rat model. The selenite-induced rat model that we adopted is a widely used screening assay for anti-cataract agents (Hiraoka and Clark, 1995). In accordance with previous studies showing that intraperitoneal injection of AKBA could activate the Nrf2/HO-1 pathway in the brains of mice, AKBA was administered to the rats by intraperitoneal injection (Wei et al., 2020)**.** The present study demonstrated that selenite induces severe histopathological changes in the lenses of selenite-induced cataract rats. Notably, we detected vacuoles, absence of anterior epithelial cells and liquefaction of lens fibers in the cataract lens. Furthermore, administration of AKBA attenuated the development of cataracts and pathological changes. Immunohistochemical staining of tissue sections revealed that the expression of Nrf2 was activated by AKBA treatment in lens epithelial cells *in vivo*.

**FIGURE 7:**
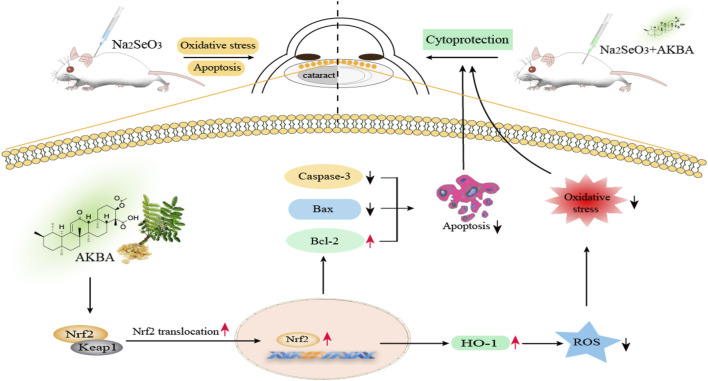
Schematic illustration of Keap1/Nrf2/HO-1 signaling activator acetyl-11-keto-beta boswellic acid (AKBA)-mediated antioxidative protection in age-related cataract. AKBA protects HLECs against H_2_O_2_-induced apoptosis and oxidative damage by activating the Keap1/Nrf2/HO-1 cascade. Apoptotic proteins (caspase-3 and Bax) are dramatically downregulated, and the antiapoptotic protein Bcl-2 is significantly upregulated after AKBA treatment. AKBA also alleviates H_2_O_2_-induced oxidative stress by reducing the activity of ROS. Additionally, AKBA stimulates the nuclear translocation of Nrf2, which dissociates from the Nrf2/Keap1 complex and promotes HO-1 expression. Furthermore, AKBA treatment exerts an inhibitory effect on the progression of selenite-induced cataracts in rats.

Overall, we demonstrated that AKBA could protect HLECs against H_2_O_2_-induced apoptosis and oxidative damage by activating the Keap1/Nrf2/HO-1 cascade. In addition, AKBA treatment exerted a protective effect on selenite-induced cataract development in rats by activating Nrf2 (Figure 7). Thus, the present research provides evidence that AKBA might be a potential therapeutic agent to prevent and delay ARC in the clinic.

## Data Availability

The raw data for statistical analysis and raw data of Western blot are presented in Supplementary Material.
