# Supplementary figures and images for: Acetyl-11-Keto-Beta Boswellic Acid (AKBA) Protects Lens Epithelial Cells Against H2O2-Induced Oxidative Injury and Attenuates Cataract Progression by Activating Keap1/Nrf2/HO-1 Signaling
Source: Front Pharmacol. 2022 Jul 11;13:927871. doi: 10.3389/fphar.2022.927871 (PMC9310784; doi:10.3389/fphar.2022.927871)

Figure 1F

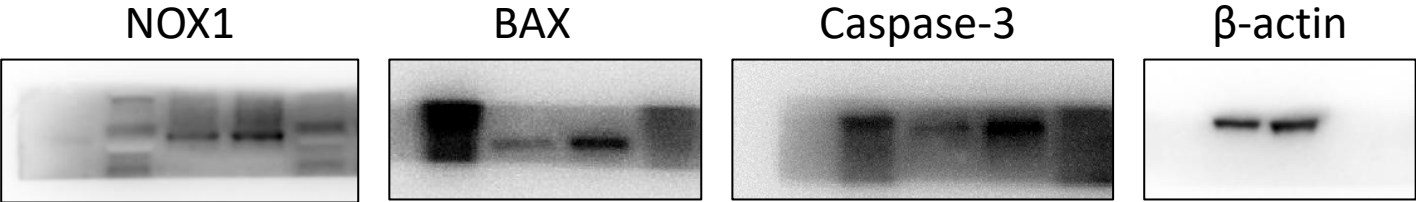

Figure 3B

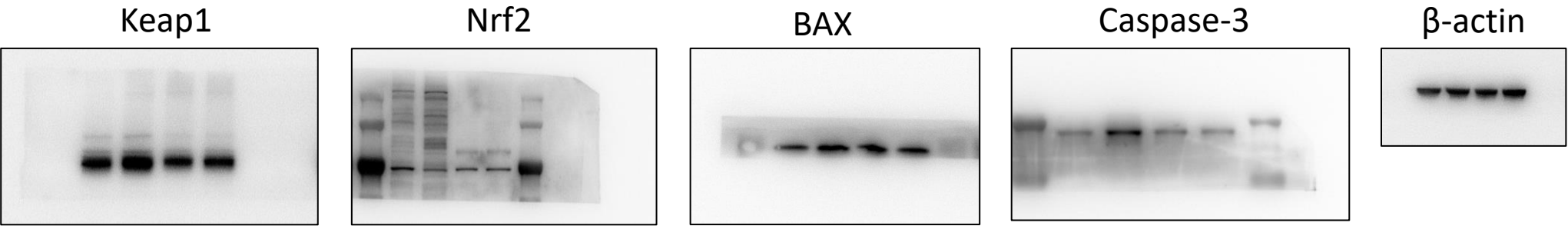

Figure 3D

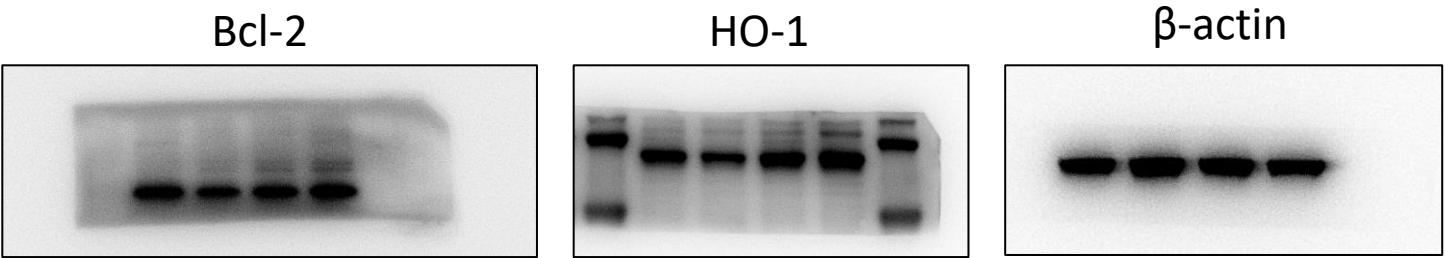

Figure 3F

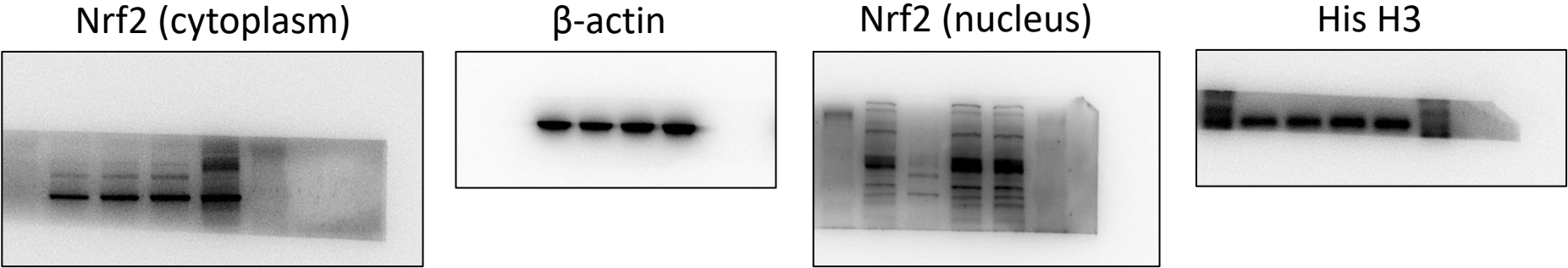

Supplement: Supplementary file 1 [file DataSheet2.pdf]
