# Supplementary material for: Acetyl-11-Keto-Beta Boswellic Acid (AKBA) Protects Lens Epithelial Cells Against H2O2-Induced Oxidative Injury and Attenuates Cataract Progression by Activating Keap1/Nrf2/HO-1 Signaling
Source: Front Pharmacol. 2022 Jul 11;13:927871. doi: 10.3389/fphar.2022.927871 (PMC9310784; doi:10.3389/fphar.2022.927871)
Supplement: Supplementary file 2 [file DataSheet3.pdf]

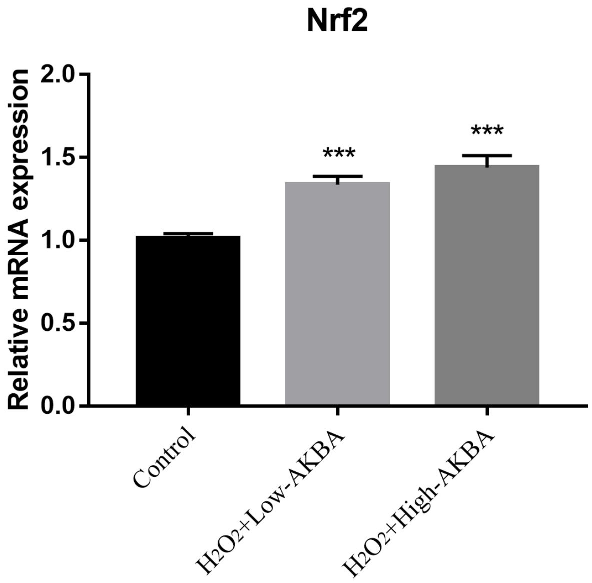

Figure R1. The mRNA expression of Nrf2 can be markedly upregulated by AKBA treatment alone. ***P<0.001, vs. the control group.
